# Microstructural Evolution and Dry Sliding Wear Behavior of a Cu-Cr-Zr Alloy Processed by Cyclic Hot Forging and Short-Time Aging

**DOI:** 10.3390/ma19163374

**Published:** 2026-08-07

**Authors:** Chenghua Gao, Ao Meng, Zihao Wang, Wei Jiang, Zhumin Li, Yu Zhao, Jiansheng Li

**Affiliations:** 1 School of Materials Science and Engineering, Anhui Polytechnic University, Wuhu 241000, China; 2250210110@stu.ahpu.edu.cn (C.G.); 2240222102@stu.ahpu.edu.cn (Z.W.);; 2 Anhui Key Laboratory of Advanced Non-Ferrous Metal Materials, Wuhu 241000, China

**Keywords:** Cu-Cr-Zr alloy, hot forging, microstructural evolution, friction and wear

## Abstract

To elucidate the effect of cyclic hot forging and short-time aging (HFSTA) on the wear resistance of a Cu-Cr-Zr alloy, samples in the as-received state (solution-treated at 1000 °C) and after 4 passes and 6 passes of cyclic HFSTA at 450 °C were prepared. The relationships between microstructure and properties were systematically analyzed. The results indicate that the cyclic HFSTA process did not change the main phase structure of the Cu matrix but significantly tailored the grain morphology, local misorientation, grain boundary character, and tribo-chemical behavior of the surface. The 4-passes sample possessed relatively high hardness, electrical conductivity, and favorable microstructural stability and was able to form a continuous and dense oxide protective film during friction, as demonstrated by the increase in hardness from 86 HV (as-received) to 195 HV, the decrease in average coefficient of friction (COF) from 0.65 to 0.50, and the reduction in wear rate from 13.3 × 10^−4^ mm^3^/(N·m) to 0.3 × 10^−4^ mm^3^/(N·m). The 6-passes sample exhibited slightly higher hardness, but the increased proportion of low-angle grain boundaries (LAGBs) intensified cracking and spallation on the wear track, causing the wear rate to rebound to approximately 8.2 × 10^−4^ mm^3^/(N·m). The study demonstrates that the wear resistance of the Cu-Cr-Zr alloy does not improve monotonically with hardness or grain refinement but is jointly controlled by precipitation strengthening, dislocation/substructure strengthening, surface damage tolerance, and the stability of the tribo-film. For the dry sliding service conditions of Cu-Cr-Zr alloys, 4 passes of cyclic HFSTA at 450 °C represent an optimal processing window that balances mechanical properties, electrical conductivity, and wear resistance, providing guidance for the process optimization of components such as contact wires and welding electrodes.

## 1. Introduction

Cu-Cr-Zr alloys combine high electrical conductivity, thermal conductivity, softening resistance, and mechanical strength, making them important copper-based alloys for resistance welding electrodes, electrified railway contact wires, and aerospace thermal management components [1,2]. Their advantageous properties stem primarily from the solution-precipitation behavior of Cr and Zr in the Cu matrix: solution treatment provides a supersaturated matrix, and subsequent aging produces fine Cr-rich or Zr-containing precipitates, thereby increasing strength and hardness with only a minor sacrifice in electrical conductivity [3]. This is because both Cr and Zr have low solid solubility in Cu and readily form fine nano-dispersed phases that strengthen the copper alloy [4]. However, under current-carrying, contact, sliding, or impact conditions, Cu-Cr-Zr alloys inevitably experience substantial friction and wear, accompanied by adhesive transfer, plowing, repeated fracture of oxide films, and fatigue delamination on the worn surface [5]. Consequently, wear has become one of the primary causes of failure in Cu-Cr-Zr alloy components.

To reduce wear rate, prolong service life, and meet the ever-growing industrial demands, the conventional strategy often regards higher hardness as the primary route to better wear resistance [6,7,8]. Grain refinement and aging treatments are typically employed to strengthen Cu-Cr-Zr alloys, thereby extending their service life and significantly improving wear resistance. For example, Purcek et al. [9] tailored the microstructure of a Cu-Cr-Zr alloy through severe plastic deformation (SPD) by high-pressure torsion (HPT) combined with aging; the hardness was doubled and the wear rate was reduced by an order of magnitude, achieving an ideal combination of high strength and high wear resistance. Zhang et al. [10] employed hot rolling and aging to obtain a fine-grained hardened matrix with uniformly dispersed nanoscale Ti and CuTi phases in a Cu-Ti alloy, increasing the hardness from 130 HV to 250 HV while reducing the wear rate by 61%, thereby demonstrating excellent wear resistance. Ma et al. [11] tailored the microstructure of a Cu-Cr-Zr-Sc alloy through multi-pass rolling followed by aging and found that the Sc-doped alloy exhibited increases in hardness of approximately 14.2% compared with the Sc-free alloy under identical processing conditions. Moreover, the wear track depth decreased from 61.7 μm to 34.9 μm (a reduction of 43.4%), indicating markedly improved wear resistance of the Cu-Cr-Zr-Sc alloy. It should be noted that the electrical conductivity decreased from 78.05% (International Annealed Copper Standard, IACS) to 63.30% IACS, showing that this process achieved significant enhancement in mechanical properties and wear resistance rather than a simultaneous improvement in all performance indicators [11]. This implies that an optimum deformation strain and aging temperature exist for Cu-Cr-Zr-based alloys. Purcek et al. [12] introduced ultrafine-grained structures and high-density dislocations through equal-channel angular extrusion (ECAE), and subsequent aging further introduced nanoscale precipitates, significantly improving the hardness and wear resistance of a Cu-Cr-Zr alloy. The study by Meng et al. on cold rolling and aging of a Cu-Cr-Zr alloy further supports this logic by demonstrating that the deformation and aging path significantly affects strength, ductility, and electrical conductivity, and that dislocations introduced by deformation can promote the nucleation of fine precipitates, which in turn pin dislocations and enhance the hardening effect [13].

The above studies indicate that thermomechanical processing can effectively improve the overall performance of Cu-Cr-Zr alloys. However, existing work has primarily focused on SPD-aging processes such as rolling, ECAE, and HPT, while relatively little attention has been paid to the cyclic coupling of multi-pass hot forging and short-time aging (termed HFSTA). The distinct feature of this process is that the high-density dislocations, sub-grain boundaries, and localized strain concentration regions introduced by hot forging provide abundant preferential sites for solute precipitation, and the fine precipitates formed after short-time aging can effectively pin and hinder dislocation motion during subsequent hot forging, thereby promoting further substructure refinement [14,15,16,17]. The study by Yang et al. on intermediate aging after cold rolling of a Cu-Cr-Zr-Hf alloy provides strong support for this mechanism [18]. Their results show that dislocations and grain boundaries produced during the first deformation step promote the formation of nano-precipitates at defect sites, and these precipitates subsequently impede the movement of dislocations and grain boundaries during the second deformation step, thereby increasing the material strength [18]. Ma et al. found that the tensile strength of a Cu-Cr-Zr alloy reached its peak after aging at 450 °C [11]. In addition, Zhou et al. observed that Cr-rich precipitates measured approximately 3.4 nm at 475 °C but coarsened to about 10.8 nm after aging at 600 °C, resulting in a reduction in strength [19]. Therefore, the short-time aging applied between deformation passes should promote early-stage precipitation while avoiding the pronounced coarsening typically caused by a single long-time aging treatment [20]. It is evident that the inter-pass short-time aging is not a conventional final aging but is designed to achieve a cyclic coupling in which deformation-generated defects promote solute precipitation and the precipitates then pin dislocations during subsequent deformation to enhance strength. Nevertheless, the influence of the cyclic coupling of multi-pass HFSTA on microstructural evolution and friction and wear behavior still needs further clarification.

Based on the abovementioned results, the present study fabricated three distinct groups of Cu-Cr-Zr alloy samples using the cyclic coupling of multi-pass HFSTA. The effects of this cyclic coupling on the microstructure and tribological properties of the alloy were systematically analyzed. Through further microstructural characterization, the wear mechanisms of the Cu-Cr-Zr alloy during friction under different passes were revealed. This study provides a theoretical basis and processing strategies for the wear-resistant design of Cu-Cr-Zr alloys.

## 2. Materials and Methods

### 2.1. Preparation of Three Distinct Groups Cu-Cr-Zr Alloys

**Figure 1:**
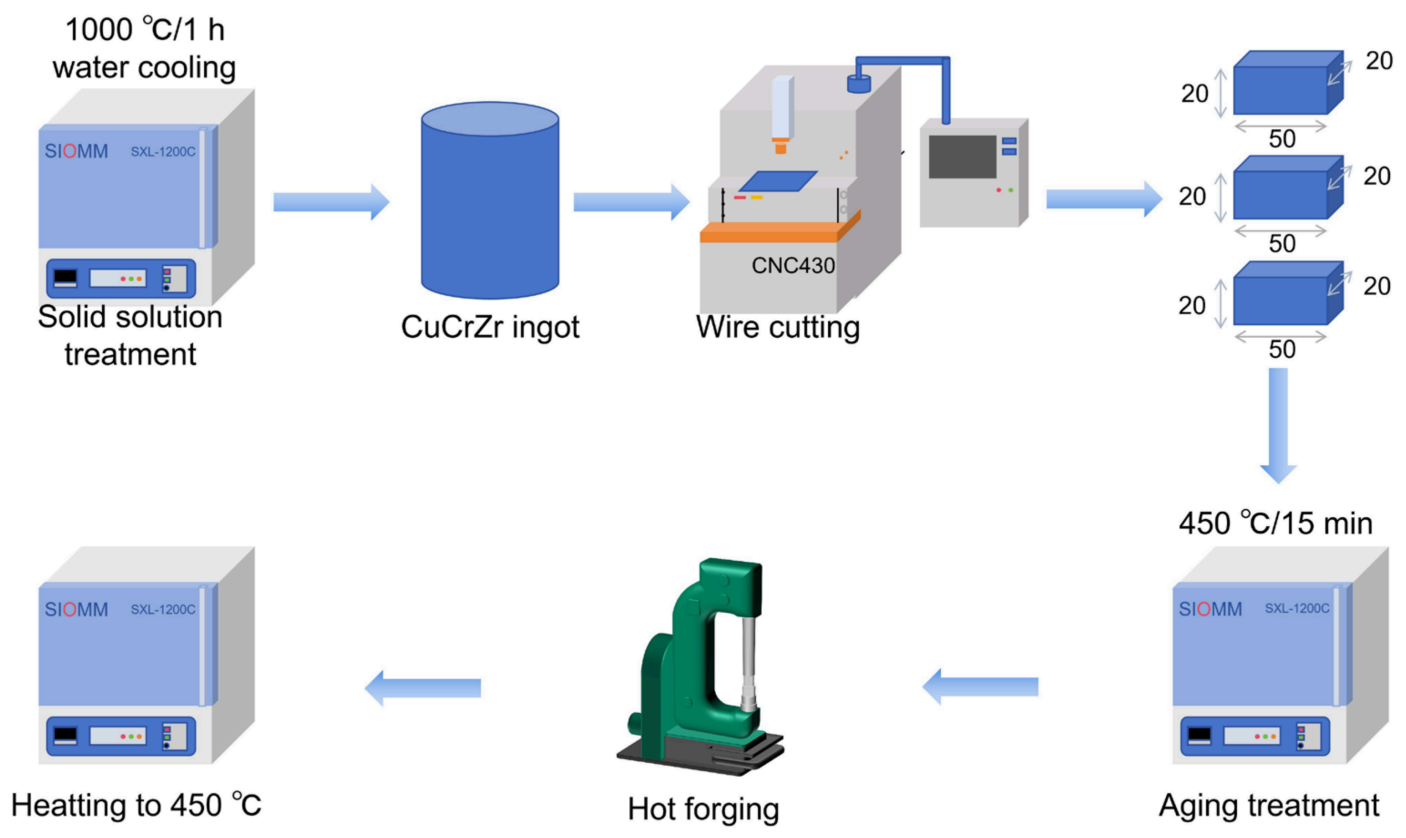
Schematic diagram of the preparation process involving cyclic hot forging and short-time aging for the Cu-Cr-Zr alloy.

The experimental material was a Cu-Cr-Zr alloy supplied by Pengda Metal Materials Co., Ltd (Shenzhen, China), and its main composition is presented in Table 1. As illustrated in Figure 1, the alloy was first solution-treated at 1000 °C for 1 h and then water-quenched to obtain the as-received sample. The solution-treated sample was subsequently cut into specimens with dimensions of 50 mm × 20 mm × 20 mm and subjected to 4-passes and 6-passes HFSTA cycles, respectively. Every single cycle consisted of heating to 450 °C for hot forging and subsequently holding at 450 °C for 15 min. The 4-passes and 6-passes samples repeated this cycle four and six times, respectively. In each pass, the billet was compressed along the height direction from 20 mm to approximately 14 mm, corresponding to a height reduction of ~30% and a true strain of ~0.36 per pass (ε = ln(h_0_/h_1_)). The corresponding strain rate, estimated from the ram speed and instantaneous specimen height, was on the order of 10^−1^ to 10^0^ s^−1^. It should be noted that the strain rate was not precisely controlled as a fixed parameter due to the use of a conventional hydraulic press; however, all samples were processed under identical conditions to ensure internal consistency. The temperature of 450 °C was chosen because it lies within the typical peak or near-peak aging regime of Cu-Cr-Zr alloys, and the 15 min short-time aging promotes the de-solvation and early precipitation of Cr and Zr while reducing the risk of precipitate coarsening [13]. For ease of subsequent description, the three groups of samples are referred to as the as-received, 4-passes, and 6-passes, respectively.

### 2.2. Hardness and Tribological Test

Vickers hardness tests were conducted using an HVS-1000 microhardness tester (Shanghai Materials Testing Machine Factory, Shanghai, China) with a load of 500 g (HV0.5) and a dwell time of 15 s. For each sample, at least ten randomly selected positions were measured, and the average value was calculated to reduce errors caused by microstructural inhomogeneity. Electrical conductivity was measured with an eddy current conductivity meter (Sigmascope SMP10, Helmut Fischer, Sindelfingen, Germany) and expressed according to the IACS. Each sample was tested at least ten times, and the average value was recorded. Dry sliding friction and wear tests were performed on an HT-1000 tribometer using a ball-on-disk rotational contact configuration with a GCr15 steel ball as the counterpart. The normal load was 10 N, the rotational speed was 31.8 r/min, the test duration was 30 min, and the test temperature was room temperature. The coefficient of friction (COF) was recorded in real time with the number of cycles. The wear volume was calculated from the three-dimensional profile of the wear track, and the wear rate was determined as W_R_ = V/(F·L), where V is the wear volume (mm^3^), F is the applied load (N), and L is the sliding distance (m). Prior to all tests, the specimen surfaces were mechanically ground, polished, and cleaned to minimize the influence of initial surface roughness variations.

### 2.3. Characterization

X-ray diffraction (XRD) analysis was performed to examine the phase structures and preferred orientation variations in the samples processed under different conditions. XRD patterns were collected on a SmartLab X-ray diffractometer (Rigaku, Tokyo, Japan) using Cu Kα radiation at a tube voltage of 40 kV and a tube current of 50 mA, with a scanning range of 20°–100°, a step size of 0.02°, and a scanning speed of 3°/min. Electron backscatter diffraction (EBSD) analysis was carried out using an Aztec Crystal system (Oxford Instruments, London, UK) with a step size of 0.3 μm. The scanned area comprised 499 × 499 measurement points, and the working distance (WD) was 14 mm. Scanning electron microscopy (SEM) and energy-dispersive spectroscopy (EDS) were performed on a Sigma 360 microscope (ZEISS, Heidenheim, Germany) equipped with an EDS system (Oxford Instruments, London, UK) to observe the worn surfaces, transfer layers, and element distributions on the counterpart surfaces. SEM imaging was conducted at an accelerating voltage of 15 kV using an SE2 detector in high-vacuum mode, with a working distance of approximately 8.1 mm. X-ray photoelectron spectroscopy (XPS) analysis of the worn surfaces was performed on a Nexsa G2 spectrometer (ThermoFisher Scientific, Waltham, MA, USA) to investigate the chemical states of Cu and O. High-resolution spectra of Cu 2p (925–970 eV) and O 1s (525–545 eV) were acquired for analyzing the chemical states of Cu and O.

## 3. Results and Discussion

### 3.1. Microstructure Analysis of the Cu-Cr-Zr Alloys

**Figure 2:**
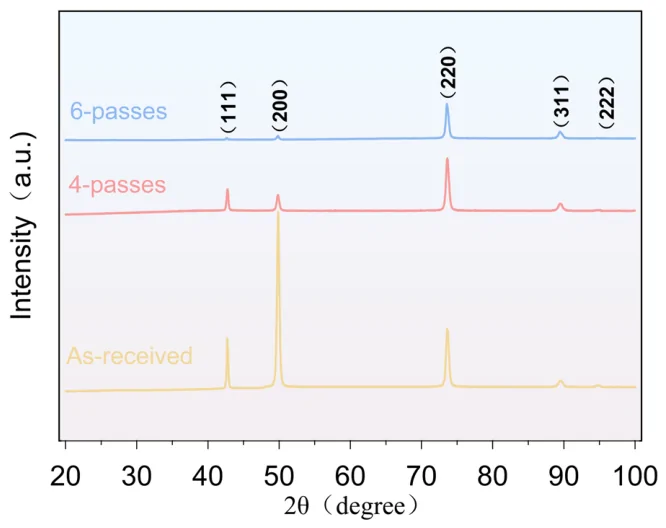
XRD patterns of the as-received, 4-passes, and 6-passes Cu-Cr-Zr alloys.

Figure 2 shows that all three samples exhibit typical diffraction peaks of a face-centered cubic (FCC) Cu matrix, with the main peaks corresponding to the (111), (200), (220), (311), and (222) crystal planes. No obvious new phase peaks of Cr, Zr, or coarse oxides are detected at the current resolution, indicating that the hot forging-aging cycle does not alter the primary phase structure of the alloy [21]. If Cr-rich or Zr-containing nanoprecipitates are present, their low volume fraction and small size make it difficult to form independent strong peaks in conventional XRD [22,23]. For example, Jiao et al. [22] observed only FCC-Cu matrix peaks in a Cu-Cr-Zr-Ni-Si alloy by XRD, while TEM confirmed the existence of nano-sized Cr precipitates approximately 3 nm in size. Given the low Cr and Zr contents of the alloy, the relatively short aging duration, and the nanoscale precipitates confirmed by Ma et al. [11], the absence of Cr/Zr second-phase peaks in the XRD patterns of the HFSTA-ed samples in this work does not rule out the possibility of Cr/Zr precipitation [23]. The authors believe that dislocations and defects introduced by deformation provide heterogeneous nucleation sites for nanophase precipitation, thereby promoting precipitate refinement. The dislocations, subgrain boundaries, and defects introduced by hot forging may facilitate the nanoprecipitation of Cr/Zr atoms [14]. It is noted that TEM work is recommended for our future study (detailed investigation is required) to directly observe the precipitate size and its interface with the Cu matrix. Compared with the as-received samples, the relative peak intensities of the 4-passes and 6-passes samples show noticeable changes, indicating that the hot forging process induces grain rotation and texture rearrangement [24]. The (200) peak is relatively prominent in the as-received sample, suggesting the presence of a certain preferred orientation after solution treatment. After hot forging and aging, the intensity distribution tends to be redistributed, reflecting the combined effects of hot deformation, dynamic recovery/recrystallization, and short-time aging [25]. These results provide a phase-structure basis for the elongated grain morphology, local misorientation changes, and grain boundary type transformations observed in the subsequent EBSD analyses.

**Figure 3:**
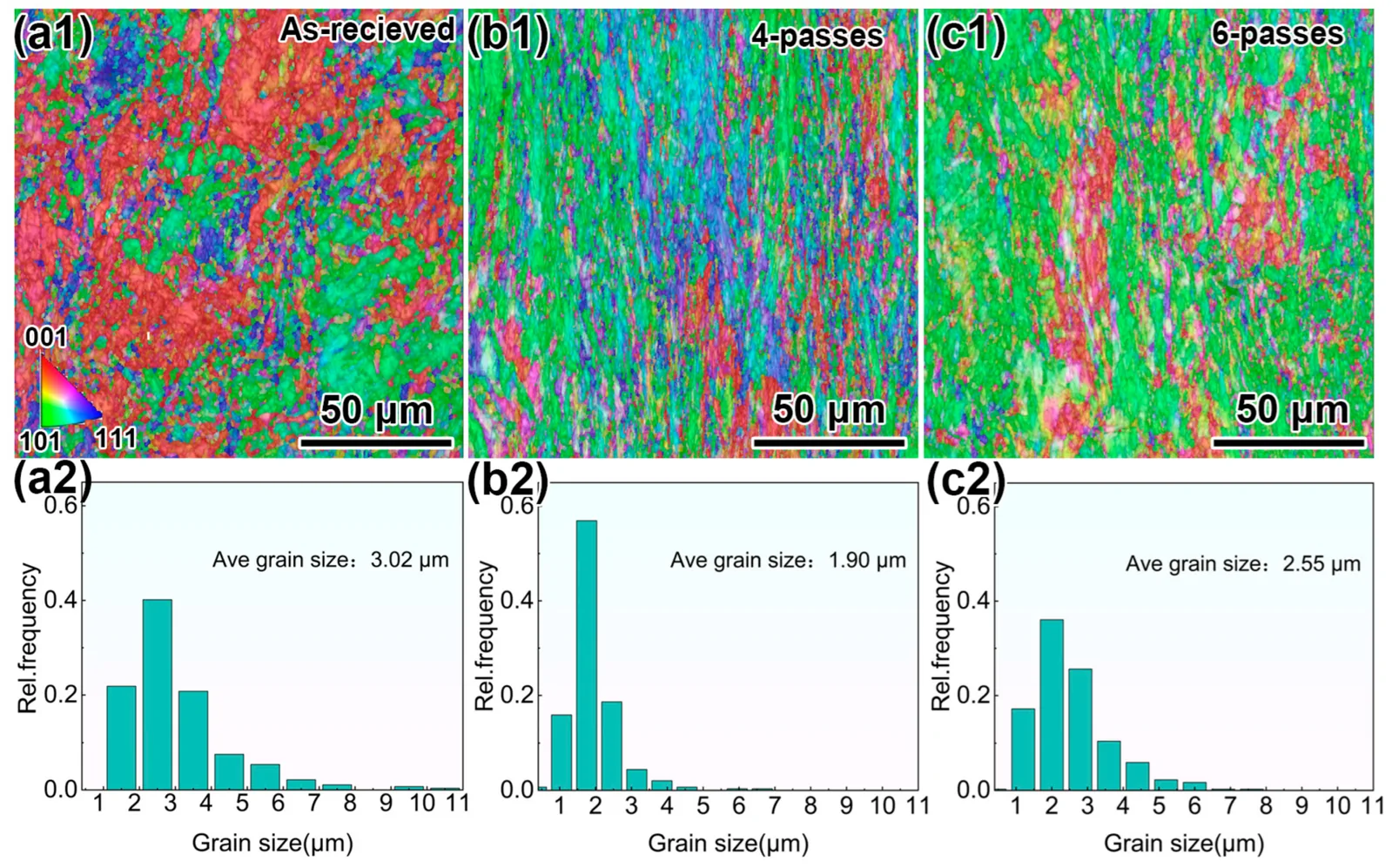
IPF maps and grain size distributions of the three Cu-Cr-Zr alloy samples: (**a1**,**a2**) as-received, (**b1**,**b2**) 4-passes, (**c1**,**c2**) 6-passes.

To further clarify the microstructure of the three samples, Figure 3 presents the inverse pole figure (IPF) maps and grain size distributions of the samples processed with different numbers of passes. In the as-received sample (Figure 3(a1)), the color variation in the grains is relatively dispersed, displaying relatively randomly oriented equiaxed grains together with local coarse-grain regions, which indicates that the microstructure after solution treatment still retains some coarse grains and non-uniform orientation. The statistical analysis in Figure 3(a2) shows that the grain size is predominantly concentrated in the small-size range, although a small number of larger grains still exist. As a result, the average grain size is 3.02 μm and the maximum grain size reaches 10.56 μm. After 4 passes of HFSTA (Figure 3(b1)), the grains become clearly elongated and banded along the deformation direction, indicating that hot forging induces grain rotation, subgrain formation, and deformation band development. Meanwhile, the continuous local color variation suggests that the sample exhibits a preferred orientation. This arises because the deformation of copper alloys primarily proceeds via {111} <110> slip systems, leading to lattice rotation [26]. The number of small-sized grains remains dominant in Figure 3(b2), and the average grain size decreases to 1.90 μm, while the maximum grain size is 6.36 μm. Figure 3(c1) demonstrates that the grains are further elongated in the 6-passes sample, with more pronounced deformation texture and stronger orientation banding in the color map, which is consistent with the XRD results (Figure 2). As shown in Figure 3(c2), the average grain size is 2.55 μm and the maximum grain size is 7.48 μm.

**Figure 4:**
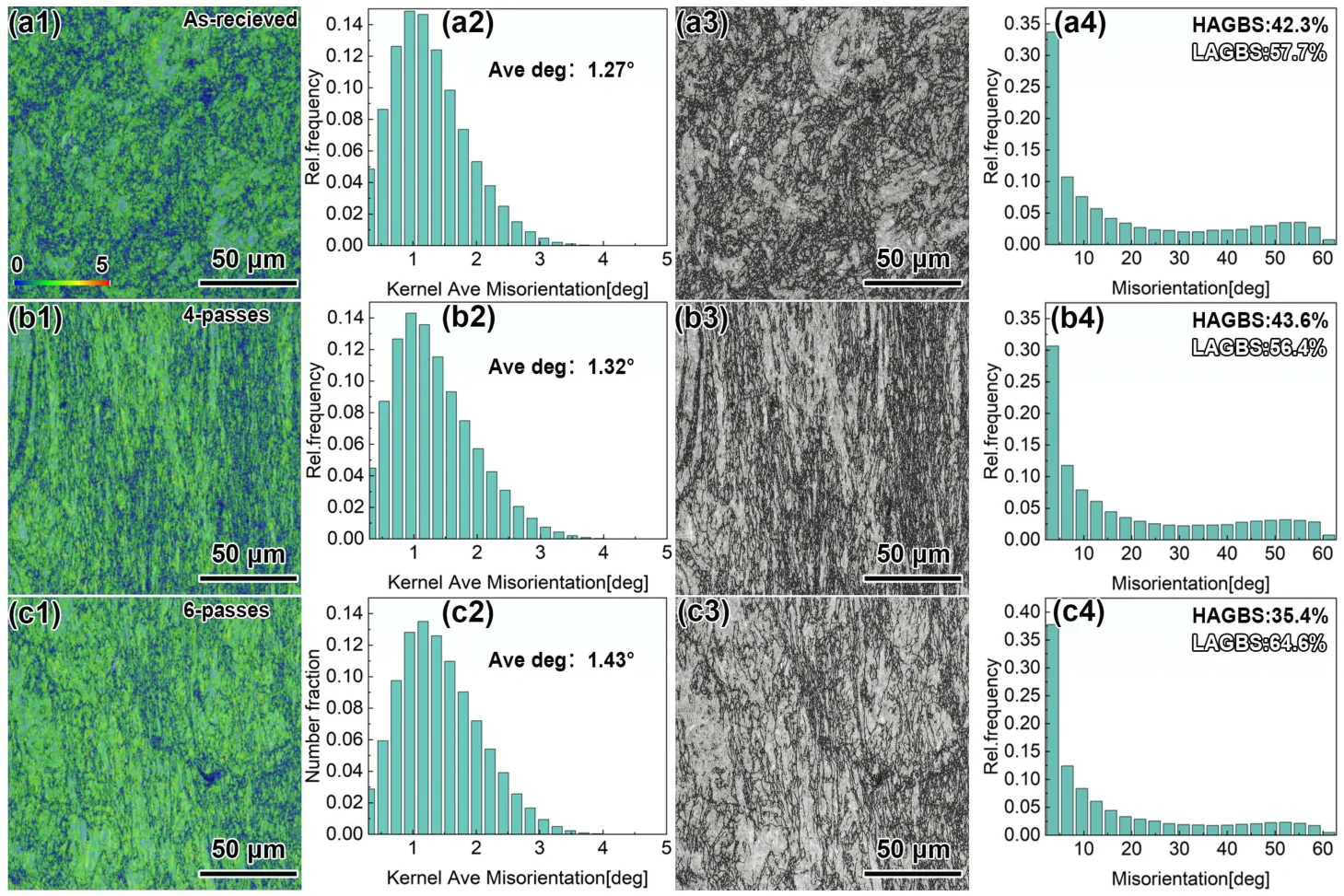
KAM, local misorientation, grain boundary, and misorientation distribution maps of the three Cu-Cr-Zr alloy samples: (**a1**–**a4**) as-received, (**b1**–**b4**) 4-passes, (**c1**–**c4**) 6-passes. It should be noted that in the misorientation distribution maps, black lines represent high-angle grain boundaries, while white lines represent low-angle grain boundaries.

The KAM maps and grain boundary distributions in Figure 4 further reveal the differences in local plastic strain and dislocation storage among the three samples. In the as-received sample, the average KAM value is 1.27°, yet some local regions exhibit high KAM values, indicating that a certain amount of residual strain is retained after solution treatment and cooling (Figure 4(a1,a2)). The KAM distribution of the 4-passes sample is similar to that of the as-received, but its average KAM value increases to 1.37°, suggesting that the dislocations introduced by hot forging exceed those annihilated by dynamic recovery/recrystallization (Figure 4(b1,b2)). The grain boundary distribution maps show that the as-received sample contains 42.3% high-angle grain boundaries (HAGBs) and approximately 57.7% LAGBs. In the 4-passes sample, the fraction of HAGBs increases to 43.6%, while that of LAGBs decreases to 56.4%. An increased proportion of HAGBs generally implies the presence of more recrystallized grains or effective grain boundaries, which helps to suppress the continuous propagation of localized shear bands and improves the stability of the surface layer during wear [27]. The 6-passes sample exhibits an average KAM value of 1.43°, a reduction in the fraction of HAGBs to approximately 35.4%, and an increase in LAGBs to approximately 64.6% (Figure 4(c1,c4)). This indicates that excessive HFSTA cycles lead to the accumulation of dislocations, subgrain boundaries, and incompletely transformed deformation substructures. LAGBs and high-density dislocations can increase hardness, but they may also serve as pathways for crack initiation and shear localization under cyclic frictional loading [28].

Considering the grain sizes of the three samples, it is evident that the grain size does not significantly decrease with increasing HFSTA cycles. The grain refinement and microstructural evolution during the multi-pass HFSTA process can be rationalized in terms of continuous dynamic recrystallization (CDRX). In CDRX, grain refinement proceeds through the progressive increase in misorientations of strain-induced low-angle boundaries, which gradually evolve into high-angle boundaries with increasing cumulative strain [29,30]. This mechanism has been well documented in copper alloys subjected to large-strain deformation, including Cu-Cr-Zr alloys [31]. From the as-received state to 4 passes, the deformation introduced dislocation substructures and sub-grain boundaries; through CDRX, a portion of the LAGBs transformed into HAGBs, leading to grain refinement (from 3.02 µm to 1.90 µm) and a slight increase in HAGB fraction (from 42.3% to 43.6%). However, from 4 passes to 6 passes, two competing processes occurred. Further deformation introduced new dislocation substructures and LAGBs within the existing grains. Meanwhile, the elevated temperature and cumulative strain promoted dynamic recovery and some grain boundary migration. The net result was that while some grains grew slightly (to 2.55 µm), the additional deformation also generated a significant number of new LAGBs within the coarser grains, effectively diluting the overall HAGB fraction to 35.4%. This interpretation is consistent with the finding that in CDRX, the transformation of LAGBs to HAGBs does not necessarily proceed monotonically with continued deformation, as newly introduced deformation substructures continuously replenish the LAGB population. It should be acknowledged that while the present EBSD observations are consistent with the CDRX mechanism, direct evidence such as detailed local misorientation analysis or in situ characterization would further strengthen this interpretation and will be pursued in future work.

### 3.2. Hardness and Electrical Conductivity

Table 2 summarizes the hardness and electrical conductivity of the three Cu-Cr-Zr alloys. As the number of HFSTA passes increased, the grain size did not continue to refine as expected but showed a trend of coarsening. Nevertheless, the hardness rose from 86 HV in the as-received state to 195 HV after 4 passes and further to 201 HV after 6 passes. The electrical conductivity increased from approximately 30.7% IACS in the as-received state to 47.8% IACS after 4 passes, reaching 52.3% IACS after 6 passes. This indicates that the hardness improvement cannot be explained by grain size alone. Grain coarsening weakens part of the Hall-Petch grain-boundary strengthening, yet the hot forging with short-time aging simultaneously enhances dislocation/substructure strengthening and precipitation strengthening, which can offset the softening caused by grain growth. Zhou et al. [19] verified this in a strengthening model for Cu-Cr-Zr alloys, where they divided the strength contributions into solid-solution, grain-boundary, dislocation, and precipitation strengthening. In their work, grain-boundary strengthening contributed only about 15 MPa, whereas the precipitation strengthening from Cr-rich precipitates after aging reached as high as 433.9 MPa, far exceeding the grain-boundary contribution [19]. During aging, Cr and Zr deplete from the Cu matrix to form GP zones, Cr-rich nano-precipitates, or Zr-rich related phases, which pin dislocation motion and provide strengthening [15]. The increase in electrical conductivity observed in the 4-passes and 6-passes samples serves as indirect evidence that Cr/Zr solute atoms have precipitated, thereby reducing electron scattering and raising the conductivity [13]. In addition, a stable Cu_5_Zr phase can be formed during prolonged aging [32]. In the present alloy, the Cr/Zr weight ratio is ~10.9, well above the reported threshold of ~3.5 for Cu_5_Zr-dominated precipitation [33]. Thus, Cr-rich precipitates are expected to be the primary strengthening phase. However, given the short aging time (15 min/pass), Zr-containing precipitates are more likely to exist as early-stage Zr-rich atomic clusters rather than fully developed Cu_5_Zr. Moreover, the high-density dislocations, dislocation cells, and LAGBs introduced by hot forging can strongly interact with nano-sized precipitates, leading to further strength enhancement [22]. Therefore, with increasing pass number, precipitates emerge in the present Cu-Cr-Zr alloy, improving both its hardness and electrical conductivity. It should be acknowledged that direct TEM observation of the Cr- and Zr-rich nano-precipitates was not performed in the present study. Future work employing TEM and related techniques will be conducted to directly visualize these precipitates and quantify their size, distribution, and chemical composition.

### 3.3. COF and Wear Rate

**Figure 5:**
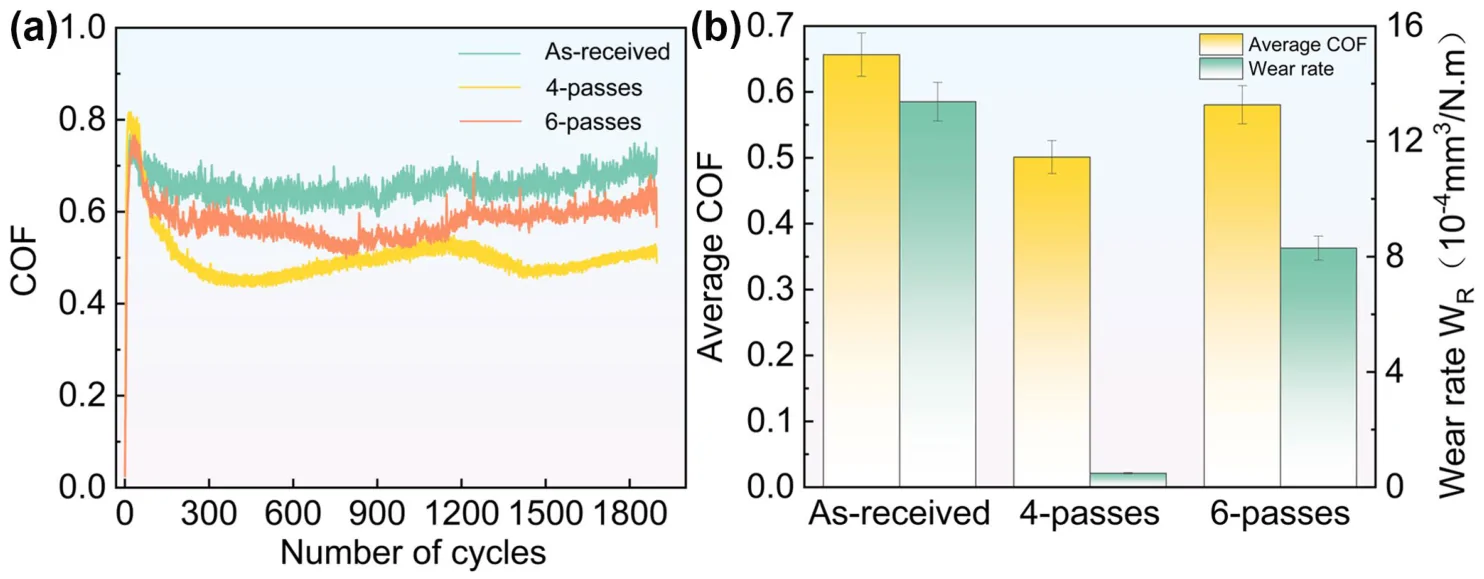
Friction and wear performance of the three Cu-Cr-Zr alloy samples: (**a**) variation in COF with the number of cycles, (**b**) average COF and wear rate.

Figure 5a presents the COF curves of the three Cu-Cr-Zr alloy samples, all of which exhibit an initial running-in stage followed by a relatively steady wear stage. For the as-received sample, the COF remained within approximately 0.62–0.72 after running-in and showed a slow upward trend over time, indicating that surface roughening, adhesive transfer, and abrasive plowing persisted during wear and that a stable protective film was difficult to form. In the 4-passes sample, the COF decreased rapidly after running-in and stabilized at around 0.45–0.55, with relatively small overall fluctuation. This suggests that the surface of the 4-passes sample possessed better shear resistance and tribo-layer stability, and that the oxide film formed during wear could reduce direct metallic contact and mitigate adhesive wear [34]. The COF of the 6-passes sample fell between those of the as-received and 4-passes samples, with a steady-stage value of approximately 0.55–0.65. Its fluctuation was more pronounced than that of the 4-passes sample, implying the involvement of local cracks, spalling pits, or wear debris in the friction process, which led to periodic destruction and reformation of the tribo-interface [35]. Figure 5b compares the average COF and wear rate of the samples processed with different numbers of passes. The as-received sample exhibited an average friction coefficient of 0.65 and a wear rate of approximately 13.3 × 10^−4^ mm^3^/(N·m). For the 4-passes sample, the average COF decreased to 0.50 and the wear rate approached 0.3 × 10^−4^ mm^3^/(N·m), demonstrating a substantial improvement in wear resistance. The average COF of the 6-passes sample was about 0.58, which was lower than that of the as-received sample but higher than that of the 4-passes sample, and its wear rate was approximately 8.2 × 10^−4^ mm^3^/(N·m), significantly higher than that of the 4-passes sample.

To further validate the significance of our findings, a quantitative comparison was made between the wear performance obtained in this study and those reported in the literature for Cu alloys under dry sliding conditions (Table 3). The 4-passes sample achieved a wear rate of 0.3 × 10^−4^ mm^3^/(N·m), which is markedly lower than that of solution treated Cu-Cr-Zr (10.0 × 10^−4^ mm^3^/(N·m)) [9], anneal-processed Cu-Zr (7.2 × 10^−4^ mm^3^/(N·m)) [36], and even the Cu-Zr alloy processed by 8-passes EACP (4.0 × 10^−4^ mm^3^/(N·m)) [36]. Moreover, the wear rate is comparable to that of the Cu-Cr-Zr alloy after undergoing HPT deformation (0.2 × 10^−4^ mm^3^/(N·m)), which represents one of the best-reported wear-resistant Cu-Cr-Zr-based alloys [9]. On the one hand, this corresponds to a ~23.1% reduction in COF compared to the as-received state, substantially outperforming the ~7.3% reduction reported for ECAP-processed Cu-Zr [36]. On the other hand, the reduction in wear rate of the 4-passes sample (~97.7%) is comparable to that of HPT-processed CuCrZr (~98%) [9]. These quantitative comparisons confirm that the 4-passes HFSTA process at 450 °C represents a highly effective thermomechanical route for achieving exceptional wear resistance in Cu-Cr-Zr alloys, competitive with or superior to many advanced processing methods reported to date.

**Figure 6:**
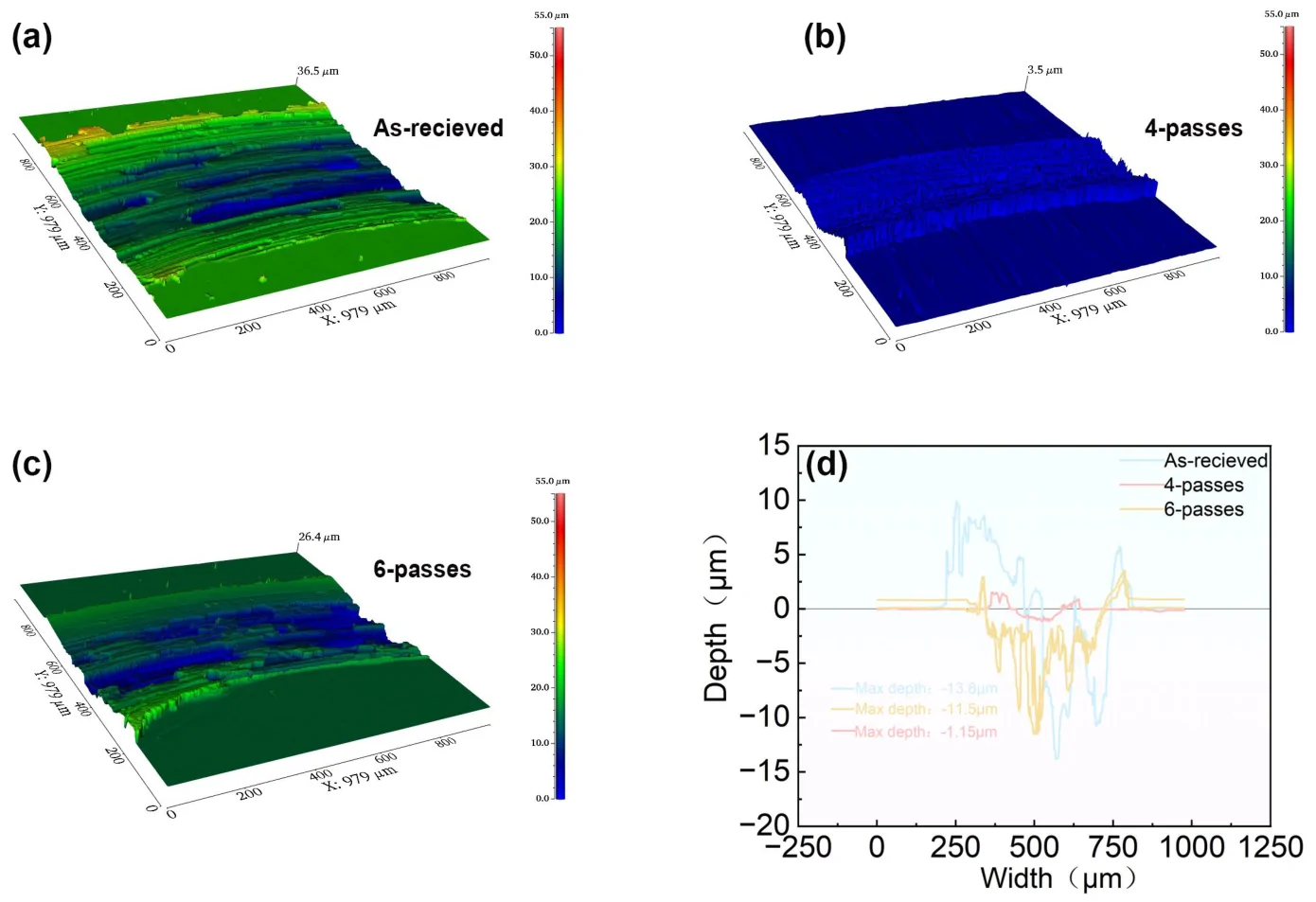
Three-dimensional wear track morphologies and cross-sectional profiles of the three Cu-Cr-Zr alloy samples: (**a**) as-received, (**b**) 4-passes, (**c**) 6-passes, and (**d**) comparison of cross-sectional depths and widths.

The three-dimensional morphology and cross-sectional profiles of the wear tracks for the three samples after testing are shown in Figure 6. Figure 6a,d reveal that the as-received sample produced a wide and deep wear track with distinct grooves, ridges, and localized material accumulation, and the maximum wear depth in the cross-sectional profile was approximately 13.8 μm. This corresponds to the high wear rate and indicates that the as-received microstructure possessed insufficient load-bearing capacity and was prone to severe plastic plowing and adhesive tearing. Figure 6b,d show that the 4-passes sample exhibited the shallowest wear track, with a maximum cross-sectional wear depth of only about 1.2 μm, a relatively flat wear track bottom, and limited material pile-up at the edges. Combined with the results in Figure 5, it can be inferred that a stable and continuous tribo-layer formed on the 4-passes sample, shifting the wear process from severe material removal to mild wear. In Figure 6c,d, the 6-passes sample displays deep grooves and a step-like morphology on the wear track, suggesting the occurrence of fatigue cracks and flake-like spallation. Consequently, the wear depth of the 6-passes sample increased again, with a maximum cross-sectional wear depth of about 11.5 μm. Although its three-dimensional wear track depth is slightly lower than that of the as-received sample, it is far greater than that of the 4-passes sample, indicating that the microstructural strengthening in the 6-passes condition could not suppress the increase in localized spallation volume.

### 3.4. Wear Surfaces Analysis

**Figure 7:**
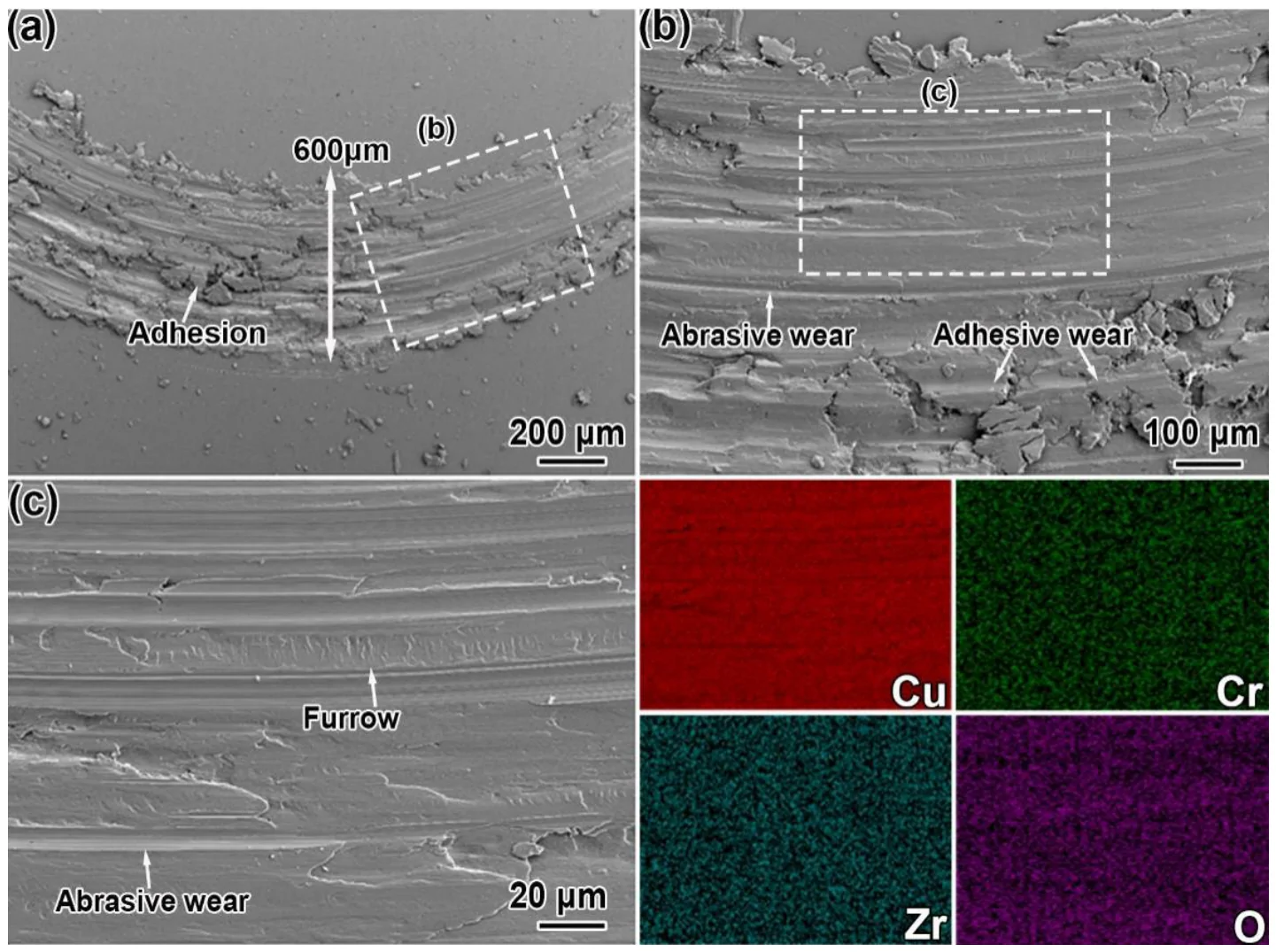
SEM morphology (**a**,**b**) of the worn surface of the as-received sample and corresponding EDS mapping (**c**).

As shown in Figure 7a, the width of the wear track is approximately 600 μm for the as-received samples, which is consistent with the three-dimensional morphology (Figure 6a). There is obvious localized spallation on the worn surface and significant material pile-up at the track edges. In Figure 7b, deep grooves along the sliding direction and scale-like spalled blocks are observed, indicating the occurrence of abrasive and adhesive wear. The EDS elemental mapping in Figure 7c reveals concentrated Cu signals in the worn area, relatively weak Cr and Zr distributions, and a certain enrichment of O in the wear track region, with an oxygen content of only 7.91 at.% (Table 4). This suggests that oxidation took place during sliding, but the oxide layer was discontinuous or insufficient to protect the substrate. It is known that three-body abrasive wear occurs when hard particles (such as fractured oxide fragments or wear debris) become entrapped between two sliding surfaces, where they are free to roll and slide, producing grooves and scratches on the worn surface [37]. The wear mechanism typically begins as two-body abrasive or adhesive wear and then gradually transitions into three-body wear due to the generation of wear debris in the form of chips or oxide particles trapped between the sliding surfaces. Purcek et al. [9] reported that the breakdown of oxide layers during sliding generates spherical wear particles, which activate the three-body abrasive wear mechanism in Cu-Cr-Zr alloys. Thus, during sliding, the fracture of the oxide layer generates spherical wear particles, which can in turn activate a new wear mechanism—three-body abrasive wear in the as-received samples.

**Figure 8:**
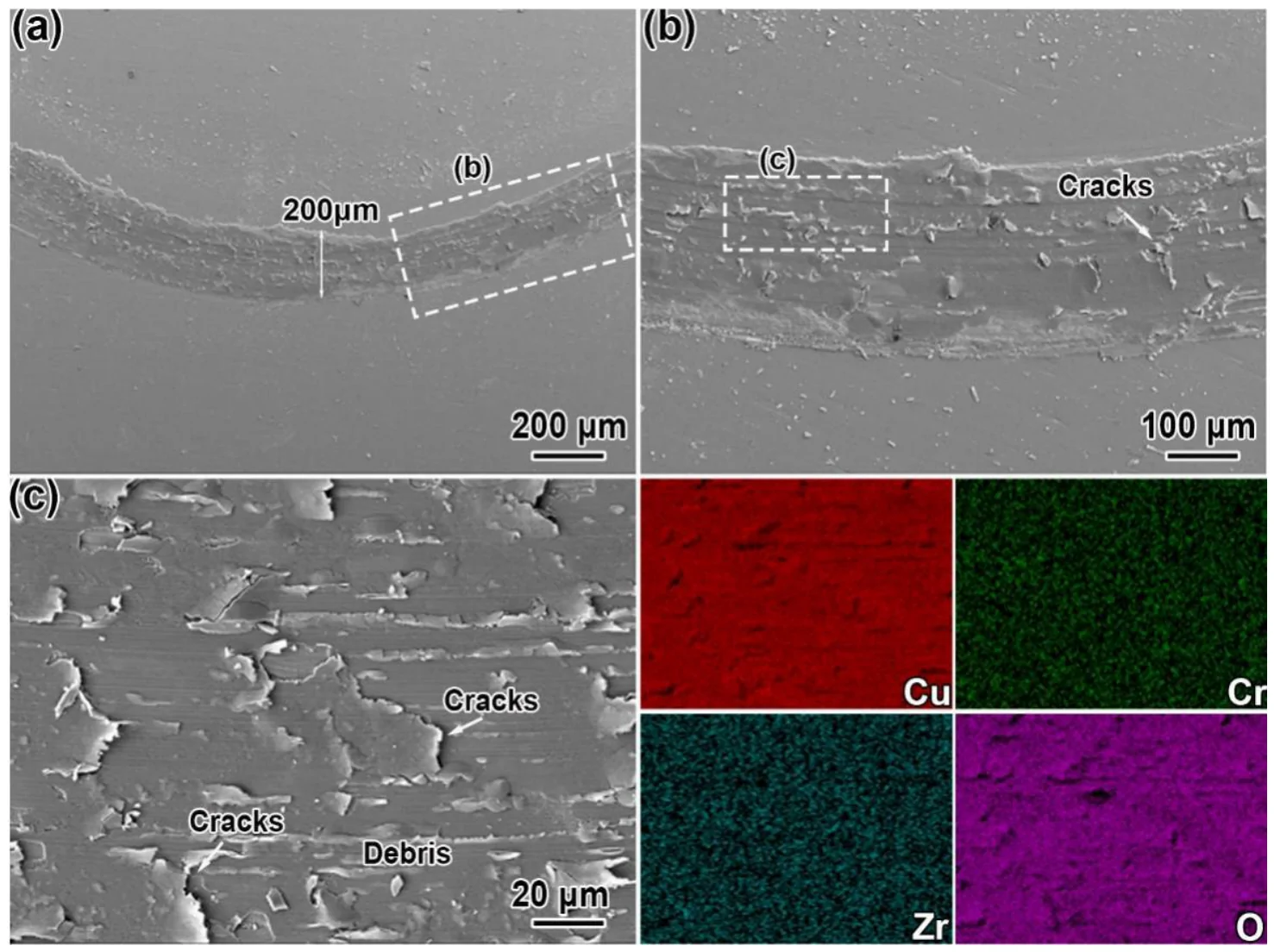
SEM morphology (**a**,**b**) of the worn surface of the 4-passes sample and corresponding EDS mapping (**c**).

As shown in Figure 8a, the wear track width of the 4-passes sample is 200 μm, which is significantly narrower than that of the as-received sample, indicating an improvement in wear resistance. Figure 8b reveals that the worn surface is relatively smooth with shallower grooves; only cracks and small debris caused by cyclic shear stress are observed, without large-area continuous spallation or the intense localized adhesive pile-up seen in the as-received sample. This indicates that the precipitation and dislocation strengthening after the 4-passes treatment enhanced the load-bearing capacity of the surface layer while retaining a certain toughness, making it difficult for cracks to propagate rapidly during the friction cycles. EDS mapping (Figure 8c) shows that Cu and O are relatively uniformly distributed in the 4-passes sample, and the O content increases to 34.80 at.% (Table 4). Therefore, it can be considered that a relatively stable and continuous oxide film formed on the 4-passes sample during sliding, which reduced direct metal-to-metal contact and suppressed adhesive wear and large-area spallation.

**Figure 9:**
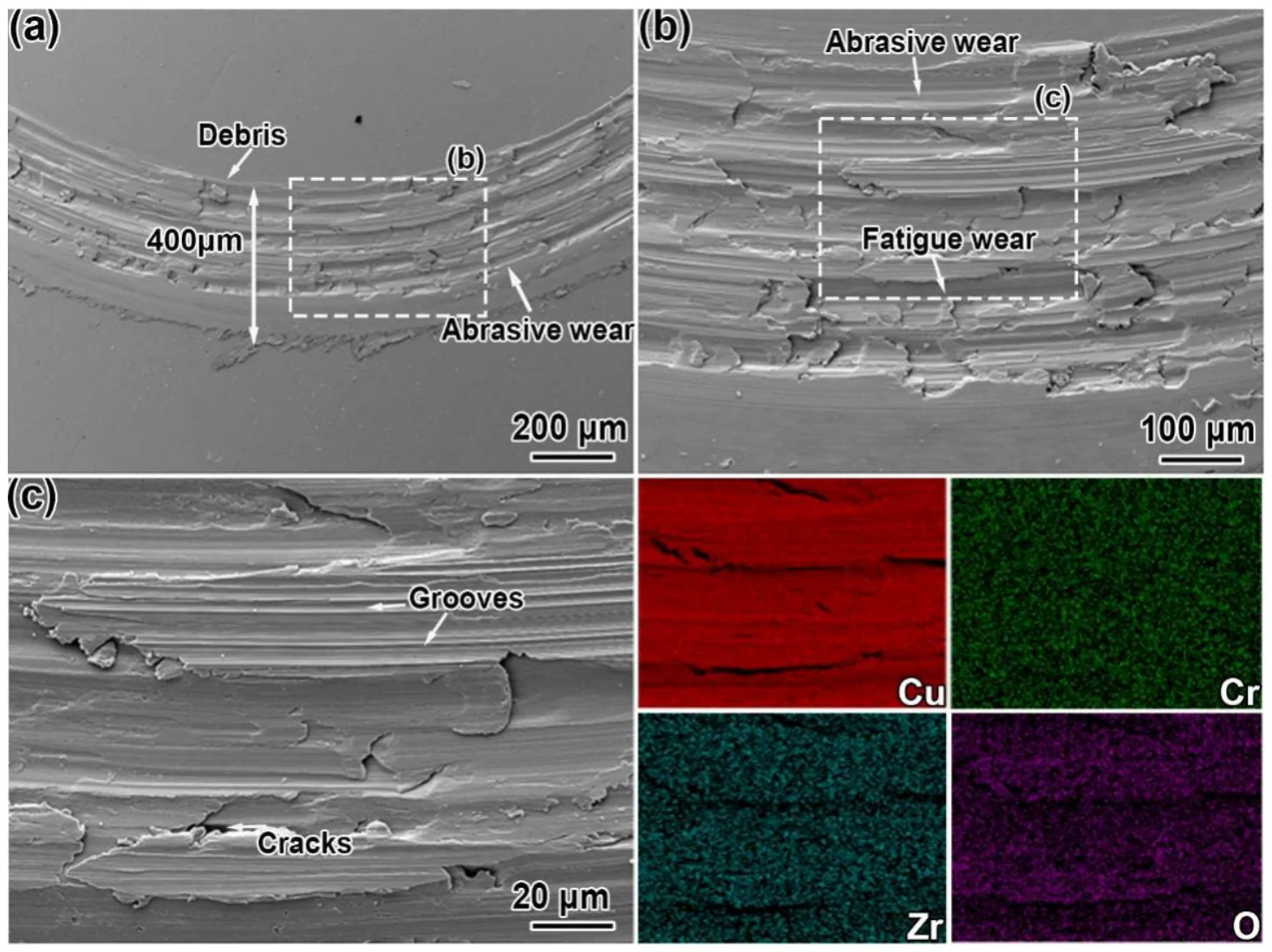
SEM morphology (**a**,**b**) of the worn surface of the 6-passes sample and corresponding EDS mapping (**c**).

Figure 9a shows that the wear track width of the 6-passes sample is approximately 400 μm, which is twice that of the 4-passes sample, and considerable debris and flake-like spallation can be observed within the track. In Figure 9b, deep grooves, cracks, and fatigue wear features are visible, which is fully consistent with the deeper wear track in Figure 6d and the rebound in wear rate shown in Figure 5b. As seen in Figure 9c, compared with the 4-passes sample, the O distribution in the 6-passes sample is less uniform, and the O content drops to 3.03 at.% (Table 4). Although excessive HFSTA cycles increased the hardness, the increased proportion of low-angle grain boundaries and substructures made the surface layer more susceptible to microcrack initiation under repeated tangential stress. After crack propagation, flake-like spallation occurs, and the spalled debris subsequently acts as third-body abrasives that promote plowing and disrupt the continuity of the oxide film. Therefore, the dominant wear mechanism of the 6-passes sample can be summarized as a coupling of abrasive wear and fatigue spallation, accompanied by adhesion and tribo-oxidation.

**Figure 10:**
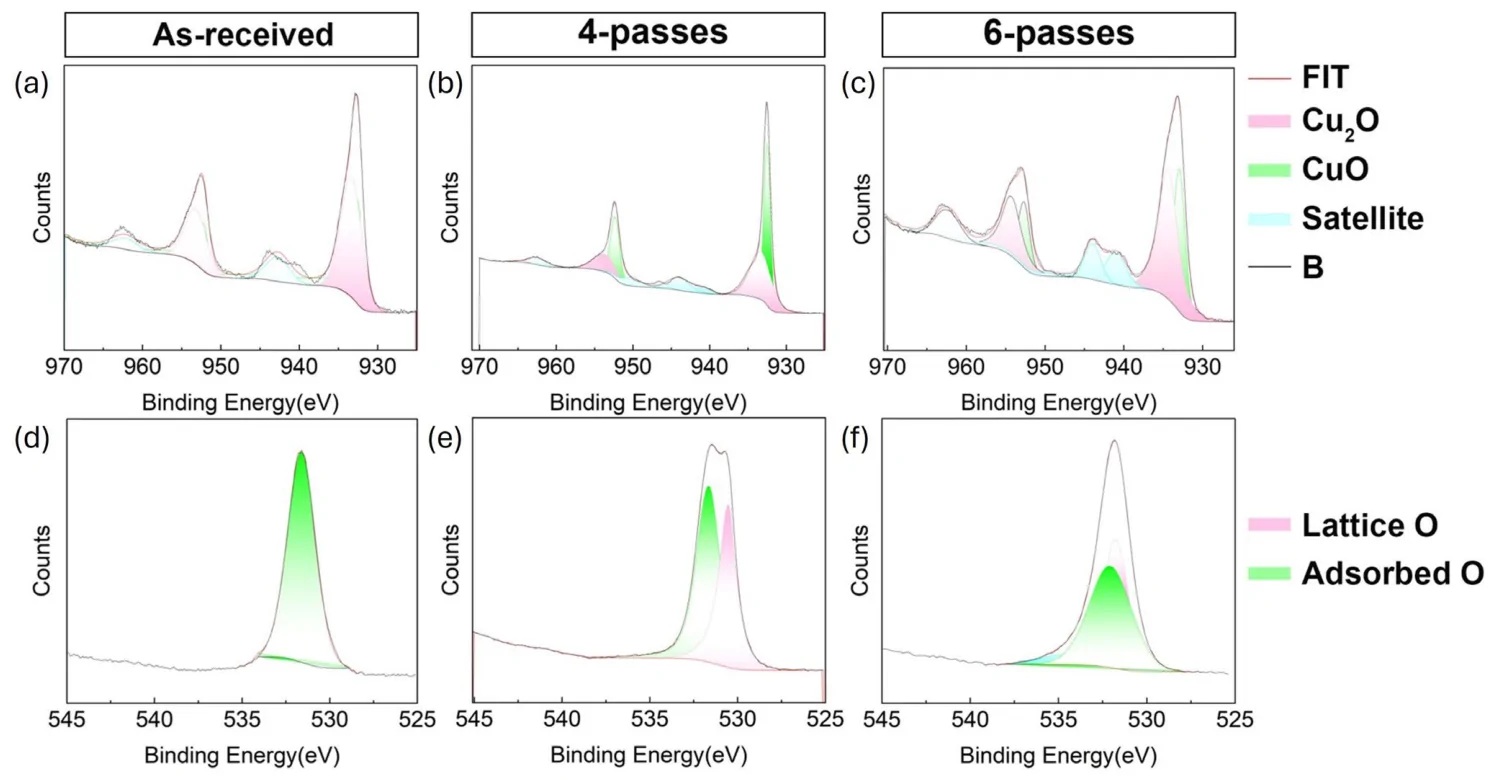
XPS analysis of the three Cu-Cr-Zr alloy samples at wear tracks: (**a**–**c**) Cu 2p spectra and (**d**–**f**) O 1s spectra.

The XPS fitting results of Cu 2p and O 1s for the worn surfaces of the three samples are presented in Figure 10. In the Cu 2p spectra (Figure 10a–c), both Cu^+^- and Cu^2+^-related components can be resolved for all samples, indicating that frictional heat generated during dry sliding promotes the reaction of freshly exposed metal surfaces with oxygen to form copper oxides. The Cu 2p satellite peaks serve mainly to identify Cu^2+^ species, whereas Cu_2_O, in which copper is in the Cu^+^ oxidation state, typically does not exhibit pronounced Cu 2p satellite peaks [38,39]. For the as-received sample, only Cu_2_O was detected on the worn surface, whereas the high-resolution Cu 2p spectra of the 4-passes and 6-passes samples (Figure 10b,c) display characteristic peaks at ~933 eV–934 eV (Cu 2p_3/2_) and ~953 eV–954 eV (Cu 2p_1/2_), together with prominent shake-up satellite peaks that are indicative of the Cu^2+^ oxidation state [40]. In addition, the O 1s spectra can be deconvoluted into a low-binding-energy lattice-oxygen component (O^2−^) and a higher-binding-energy oxygen component [41,42]. The former is generally located at approximately 529.5–530.6 eV and is related to lattice oxygen in copper oxides, whereas the latter is typically found at about 531.0–532.2 eV and is mainly associated with defect-related, hydroxyl groups (-OH), or adsorbed oxygen [15]. The as-received and 6-passes samples are dominated by the higher-binding-energy oxygen component, while the 4-passes sample exhibits both lattice-oxygen and defect-oxygen components, with a distribution closer to the low-binding-energy lattice-oxygen component, which is consistent with the formation of a relatively continuous oxide film. Based on the above results, the oxide present on the worn surface of the 4-passes sample during sliding is predominantly CuO.

### 3.5. Anti-Wear Mechanism

**Figure 11:**
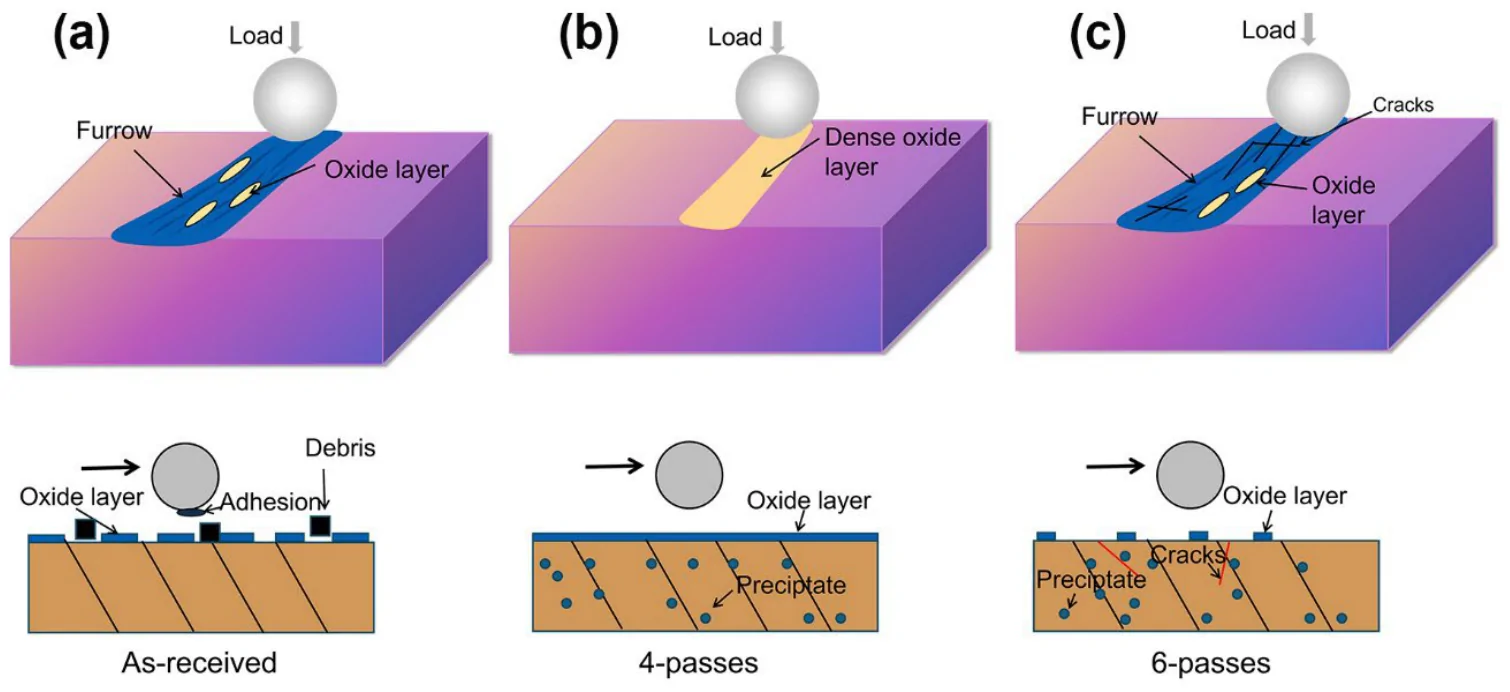
Schematic illustration of the dry sliding wear mechanisms of the three Cu-Cr-Zr alloy samples: (**a**) as-received, (**b**) 4-passes, (**c**) 6-passes.

In this work, the essential role of HFSTA for the Cu-Cr-Zr alloy is not grain refinement but rather driving the microstructure into a state more favorable for wear resistance through thermo-mechanical coupling. Hot forging is essentially high-temperature plastic deformation, which introduces dislocation tangles, LAGBs, subgrain structures, or deformation bands into the Cu-Cr-Zr matrix. These structures can impede dislocation motion and increase hardness. According to the Archard wear equation [36], increasing hardness generally helps reduce the wear volume. Chu et al. [16] pointed out that the worn surface of a low-hardness Cu-Cr-Zr sample exhibited obvious smearing, overlapping layers, and severe plastic deformation, whereas adhesive wear was significantly weakened after hardening. As shown in Figure 11a, the as-received sample possesses low hardness and insufficient surface resistance to indentation and shear, making it prone to plastic flow, adhesive transfer, and wide and deep plowing during dry sliding. Its dominant wear mechanism is adhesive-abrasive coupled wear governed by a soft matrix. The 4-passes sample shows markedly increased hardness, accompanied by the precipitation of Cr/Zr solute atoms and enhanced dislocation/substructure strengthening, which improves the surface resistance to plastic deformation and hinders plastic plowing. More importantly, a relatively continuous and stable oxide film forms on the surface, reducing direct metal-to-metal contact and suppressing adhesive wear (Figure 11b). As for the 6-passes sample, the work by Han et al. on drawing deformation of Cu-Cr-Zr indicates that increasing the deformation strain raises the proportion of LAGBs and dislocation-related interfaces [43], and these structures can act as paths for crack initiation, connection, and the formation of spallation layers under cyclic shear. This implies that although the 6-passes treatment strengthens the matrix hardness, the spalled debris and fractured oxides that enter the contact interface subsequently serve as third-body abrasives, intensifying plowing and causing the wear rate to rebound (Figure 11c). In summary, the significant differences in wear rate among the samples processed with different numbers of passes demonstrate that the dry sliding wear of the Cu-Cr-Zr alloy is not controlled solely by hardness but is jointly determined by the surface load-bearing capacity, crack sensitivity, and stability of the tribo-film.

## 4. Conclusions

Three different Cu-Cr-Zr alloy samples were prepared using multi-pass HFSTA, and the effects of HFSTA on the microstructure, hardness, and tribological properties were systematically investigated. The main conclusions are as follows:

(1) Multi-pass HFSTA can simultaneously improve the hardness and electrical conductivity of the Cu-Cr-Zr alloy. The hardness increases from 86 HV in the as-received state to 201 HV after 6 passes, and the electrical conductivity rises from approximately 30.7% IACS to about 52.3% IACS. This enhancement originates from the combined effect of dislocation/substructure strengthening and precipitation strengthening, rather than solely from grain refinement.
(2) The dry sliding wear resistance of the alloy exhibits a non-monotonic variation with increasing process passes, first improving and then deteriorating. The 4-passes sample displays the best wear resistance, with an average friction coefficient of 0.50 and a wear rate of 0.3 × 10^−4^ mm^3^/(N·m), representing a reduction of approximately 97.7% compared to the as-received sample. For the 6-passes sample, the hardness increases slightly, yet the wear rate rebounds to 8.2 × 10^−4^ mm^3^/(N·m).
(3) The non-monotonic change in wear resistance essentially reflects a trade-off between the strengthening effect and the surface damage tolerance. The 4-passes process not only enhances the matrix hardness but also maintains a relatively high fraction of HAGBs and microstructural stability, which enables the formation of a continuous and stable oxide film and suppresses plowing wear and adhesive wear. In contrast, the 6-passes process leads to a substantial accumulation of LAGBs and increased surface crack sensitivity, which intensifies fatigue spallation and third-body abrasive wear, ultimately degrading the wear resistance.

## Figures and Tables

**Table 1:** The chemical composition of the Cu-Cr-Zr alloy.

Element	Al	Cr	Fe	Mg	P	Si	Zr	Cu
**wt.%**	0.001	0.732	0.022	0.014	0.0005	0.007	0.067	Balance

**Table 2:** The hardness and electrical conductivity of the three Cu-Cr-Zr alloys.

Samples	Hardness (HV)	Electrical Conductivity (%IACS)
As-received	86	30.7
4-passes	195	47.8
6-passes	201	52.3

**Table 3:** The COF and wear rate of the Cu alloys under different conditions.

Alloy/Condition	Test Conditions	COF	Wear Rate (×10^−4^ mm^3^/(N·m))	Reference
Cu-Cr-Zr (as-received)	10 N, ball-on-disk	0.65	13.3	This work
Cu-Cr-Zr (4-passes)	10 N, ball-on-disk	0.50	0.3	This work
Cu-Cr-Zr (6-passes)	10 N, ball-on-disk	0.58	8.2	This work
Cu-Cr-Zr (solution treated)	10 N, ball-on-disk	/	10.0	[9]
Cu-Cr-Zr (HPT)	10 N, ball-on-disk	/	0.2	[9]
Cu-Zr (annealed)	10 N, ball-on-disk	0.68	7.2	[36]
Cu-Zr (ECAP-8-passes)	10 N, ball-on-disk	0.63	4.0	[36]

**Table 4:** The elemental contents (at.%) of the worn surfaces for the three Cu-Cr-Zr alloys.

Sample	Cu	Cr	Zr	O
As-received	91.08	0.77	0.24	7.91
4-passes	64.58	0.49	0.13	34.80
6-passes	95.94	0.80	0.22	3.03

## Data Availability

The original contributions presented in this study are included in the article. Further inquiries can be directed to the corresponding authors.
